# Genetically modified mesenchymal stem cells promote spinal fusion through polarized macrophages

**DOI:** 10.1038/s41374-021-00693-4

**Published:** 2021-11-11

**Authors:** Luchao Yu, Qiang Shi, Baokun Zhang, Jianguang Xu

**Affiliations:** 1 Department of Orthopedic Surgery, Shanghai Jiaotong University Affiliated Sixth People’s Hospital, Shanghai, 200233 China; 2 Department of Orthopedic Surgery, Putuo Hospital, Shanghai University of Traditional Chinese Medicine, Shanghai, 200062 China

**Keywords:** Translational research, Tissue engineering

## Abstract

Spinal fusion is an effective treatment for low back pain and typically applied with prosthetic fixation devices. Spinal fusion can be improved by transplantation of mesenchymal stem cells (MSCs) into the paraspinal muscle. However, in contrast to the direct contribution of MSCs to spinal fusion, the indirect effects of MSCs on spinal infusion have not been studied and were thus addressed here. The correlation between the outcome of spinal fusion and the local macrophage number, polarization and the levels of placental growth factor (PlGF) in patients was analyzed. MSCs were genetically modified to overexpress PlGF, and its effects on macrophage proliferation and polarization were analyzed in vitro in a transwell co-culture system, as well as in vivo in a mouse model for spinal fusion, for which the cells were bilaterally injected into paravertebral muscles of the mouse lumbar spine. The effects on spinal fusion were assessed by microcomputed tomography and a custom four-point bending apparatus for structural bending stiffness. Local macrophages were analyzed by flow cytometry. We found that posterior spinal fusion could be improved by PlGF-expressing MSCs, compared to the control MSCs, evident by significant improvement of bone bridging of the targeted vertebrae. Mechanistically, PlGF-expressing MSCs appeared to attract macrophages and induce their M2 polarization, which in turn promotes the bone formation. Together, our data suggest that PlGF-expressing MSCs may improve spinal fusion through macrophage recruitment and polarization.

## Abstract (web summary)

The authors examined the indirect effects of mesenchymal stem cells (MSCs) on spinal infusion through macrophages. They found that posterior spinal fusion is improved by placental growth factor (PlGF)-expressing MSCs, likely through attraction of macrophages and indution their M2 polarization, which in turn promotes the bone formation.

## Introduction

A great number of people who are younger than 45 years are suffered from severe low back pain, as a major reason for developing disability^1^. Most low back pain results from intervertebral disc degeneration as well as other pathological conditions such as scoliosis, spondylosis, spondylolisthesis, infection-associated inflammation, formation of cancer and posttraumatic fracture^1^. As an effective therapy of serious and sustained low back pain, spinal fusion of two or more adjacent vertebrae has been widely and routinely applied^2^.

For a successful induction of spinal fusion, bone grafts and synthetic implants have been used. However, lack of efficiency and generation of side effects render researchers to identify alternative methods, e.g., of an injectable biological material agent to promote the formation of spinal infusion without the requirement for bone grafts and synthetic implants^2^. This approach can even avoid open surgery and lengthy hospitalization, which may greatly release the burden of patients both physiologically and financially^3^.

Some pilot studies have shown promising results. For example, direct delivery of osteogenic genes such as bone morphogenetic protein-2 (BMP-2), BMP-7 and BMP-9, has been shown to facilitate spinal fusion^4–10^. Moreover, induction of spinal infusion has been also achieved through injection of mesenchymal stem cells (MSCs)^11–22^, leading to mechanical stabilization as the ultimate goal of spinal fusion^23^. MSCs are easily accessible in a non-invasive way. Moreover, MSCs possess a strong proliferative capacity to allow them to be nearly unlimitedly amplified in vitro^11–22^. However, the past studies have mainly focused on the direct contribution of MSCs to spinal fusion, while their indirect effects on spinal fusion have not been well investigated. It is noteworthy that there are a great number of evidence to show the role of MSCs in tissue repair, regeneration and remodeling relies on their crosstalk with local macrophages^24–26^. MSCs have been shown to regulate macrophage differentiation, proliferation and polarization to a phenotype called M2 (in contrast to traditional phagocytotic M1 macrophages) that are trophic and promote tissue regeneration through production and secretion of a number of growth factors^27–29^. However, the effects of MSCs on spinal fusion through macrophages have not been systemically studied.

Placental growth factor (PlGF) is a member of the vascular epithelial growth factor (VEGF) family, and plays a critical role in pathological angiogenesis^30^ and acts as a trigger for macrophage recruitment and differentiation^29,31–35^. The expression and function of PlGF in spinal fusion-associated macrophage infiltration have not been examined, and this question was thus addressed in the current study.

## Materials and methods

### Ethical approval, patient studies and experiment design

All experimental procedures have been approved by the research committee of Shanghai Jiaotong University Affiliated Sixth People’s Hospital. The clinical studies included 22 patients with a diagnosis of non-pathological thoracolumbar burst fracture in the T12-L3 range (kyphos was above 20° and/or anterior body collapse was >50%) and then received an internal fixation treatment at our hospital between 2015 and 2018. These patients included 14 male and 8 female, and the mean age at injury was 35.6 years (±11.8, range 23–49). The Levels of the fracture were T12: 7, L1: 9, L2: 5 and L3: 1. The removal of the implants was between 6 and 8 months after the surgery. The biopsy was taken at the time of the implant removal. The RNA was extracted from the biopsy and analyzed for levels of some interested genes. The patients were invited to attend a clinical and radiographic review using a Low Back Outcome Score System (LBOSS) adapted from a previous publication^36^ and using an American Spinal Injury Association (ASIA) instruction to determine the nerve injury levels both pre-operatively and post-operatively. The AISA grade (A-E) has been determined by the following rule. AISA A = no motor or sensory function is preserved in the sacral segments S4-S5. ASIA B = sensory but not motor function is preserved below the neurological level and includes the sacral segments S4-S5 ASIA C = motor is preserved below the neurological level, and most of the key muscles below the neuro level have a muscle grade <3. ASIA D = motor function is preserved below the neurological level, and at least half of key muscles below the neurological level have a muscle grade = or >3. ASIA E = NORMAL motor and sensory testing.

The mouse experiments were performed in accordance with guidance for the Care and Use of Laboratory Animals. Male and female 12-week-old C57BL/6 mice were purchased from SLAC Laboratory Animal Co. Ltd (Shanghai, China). Both male and female mice were used and distributed evenly in each experimental group. Mice were housed under a 12-hour light-dark cycle. For the in vivo mouse experiment, power calculations (*P* < 0.05) were performed to include exactly sufficient animals for the observed effects to be legitimate. An allocation concealment method was used, and efforts were made to ensure that the potential confounders were minimized. No criteria were used for excluding animals (or experimental units) during the experiment, and no data were excluded during the analysis. The study did not have humane endpoints.

### Mouse MSCs and gene editting

Mouse MSCs and naïve macrophages were isolated from bone marrow of isogeneic mice for in vivo expriments, as described in^37^ and^38^, respectively. Mouse MSCs were cultured in StemXVivo^®^ Mesenchymal Stem Cell Expansion Medium (R&D System, Los Angeles, CA, USA) and mouse macrophages were cultured in Iscove’s Modified Dulbecco’s Medium (IMDM, Thermo Scientific, Rockford, IL, USA) suppled with 15% fetal bovine serum (FBS) in an incubator at a concentration of 5% carbon dioxide at 37 °C. MSC differentiation media were purchased from American Type Culture Collection (ATCC, Rockville, MD, USA). Transfection of MSCs by a full-length mouse PlGF2 construct a scrambled sequence (scr) as a control under a CMV promoter was performed with Lipofectamine 3000 reagent (Invitrogen, St. Louis, MO, USA). The phenotype of the modified PlGF-expressing MSCs were evaluated by flow cytometry analysis of the surface markers Sca-1, CD90, CD105, CD34, CD45 and HLA-DR, and by inducible differentiation of MSCs into osteocytes, adipocytes and chondrocytes, respectively, with different differentiation media (ATCC, Catalog number: PCS-500-052, PCS-500-050 and PCS-500-051). Alcian blue staining, Von kossa staining and Oil red O staining were applied to examine the differentiated chondrocytes, osteocytes and adipocytes, respectively.

### Animal treatment and grouping

For in vivo injection of MSCs into the mouse spine, 2 × 10^6^ (modified) MSCs were suspended in 30 µl fibrin gel (FG, Sigma-Aldrich, St. Louis, MO, USA) and directly injected into the lumbar paravertebral muscle at L3-L5 after the mice were anesthetized by intraperitoneal injection of combined solution of 3 mg/kg xylazine and 100 mg/kg ketamine (Sigma–Aldrich). a blade cut of 1.5 cm long and 2 mm think was made on the middle back of the mice. For the first mouse experiment, the mice were assigned into 3 groups of 8 each: Group 1: mice received injection of MSCs-free FG; Group 2: mice received injection of FG containing MSCs-scr; Group 3: mice received injection of FG containing MSCs-PlGF. All the mice were kept for 6 weeks before analysis. For the second mouse experiment, the mice were assigned into 3 groups of 8 each: Group 1: mice received injection of FG containing MSCs-PlGF; Group 2: mice received injection of FG containing MSCs-PlGF and a weekly intravenous injection of 150 μl liposome (Clodronateliposomes, Netherlands) afterwards; Group 3: mice received injection of FG containing MSCs-PlGF and a weekly intravenous injection of 150 μl clodronate (Clodronateliposomes) afterwards. All the mice were kept for 6 weeks after the injection of FG into the lumbar paravertebral muscle.

### Co-culture of MSCs and macrophages

For the co-cultre system, transwell chambers (8 μm pore size, Corning Co., NY, USA) were sequentially added to 200 μl of cells at a concentration of 10^5 ^cells/ml and 500 μl of FBS-free culture media for macrophages. After 24 h, cells in the upper part of the small cell membrane were wiped off and determined. Arginase activity was measured with an arginase activity assay kit (MAK112, Sigma-Aldrich).

### Micro-CT analysis to assess bone formation

Specimens were fixed in paraformaldehyde for 48 h, after which microcomputed tomography

(MicroCT, mCT 40; Scanco Medical AG, Bassersdorf, Switzerland) examination was applied. Newly formed bone was separated from native bone by manual contouring. Some parameters were determined, including bone mineral density (mg hydroxyapatite (HA)/cm^3^) derived from the projectional image calculated with the calibration with H_2_KPO_4_ (Sigma–Aldrich) and the average bone thickness in micrometers. The lumbar spine rigidity was assessed by a four-point bending assay as described^23^.

### Quantitative RT-PCR and ELISA

The quantitative RT-PCR assay (RT-qPCR) was done routinely. Total RNA was extracted by a RNeasy kit (Qiagen, Valencia, CA, USA). Total RNA is transcribed by reverse transcription kit to generate cDNA for RT-qPCR (Qiagen). All primers were purchased from Qiagen. GAPDH was used as an internal control for relative gene expression.

### Flow cytometry

For flow cytometric analysis, macrophages and M2 macrophages were detected by F4/80+ and F4/80 + CD206 + , respectively. The analysis of the surface markers of the modified PlGF-expressing MSCs used fluorescin-conjugated Sca-1, CD90, CD105, CD34, CD45 and HLA-DR antibodies (Becton-Dickinson Biosciences, San Jose, CA, USA). The proliferating cells were determined by analysis with a Ki-67 antibody (Thermo Scientific, San Jose, CA, USA) on fixed cells. Data were analyzed and presented by FlowJo software (Version 10, Flowjo LLC, Ashland, OR, USA).

### Statistical analysis

All values represent the mean ± standard deviation (SD). Statistical analysis of group differences was carried out using a one-way analysis of variance (ANOVA) test (GraphPad prism 7, GraphPad Software, Inc. La Jolla, CA, USA) followed by the Fisher’s Exact Test to compare two groups. A value of *p* < 0.05 was considered significant.

## Results

### Patient data suggest a possible role of macrophages in the recovery of the internal fusion after thoracolumbar fracture

**Fig. 1:**
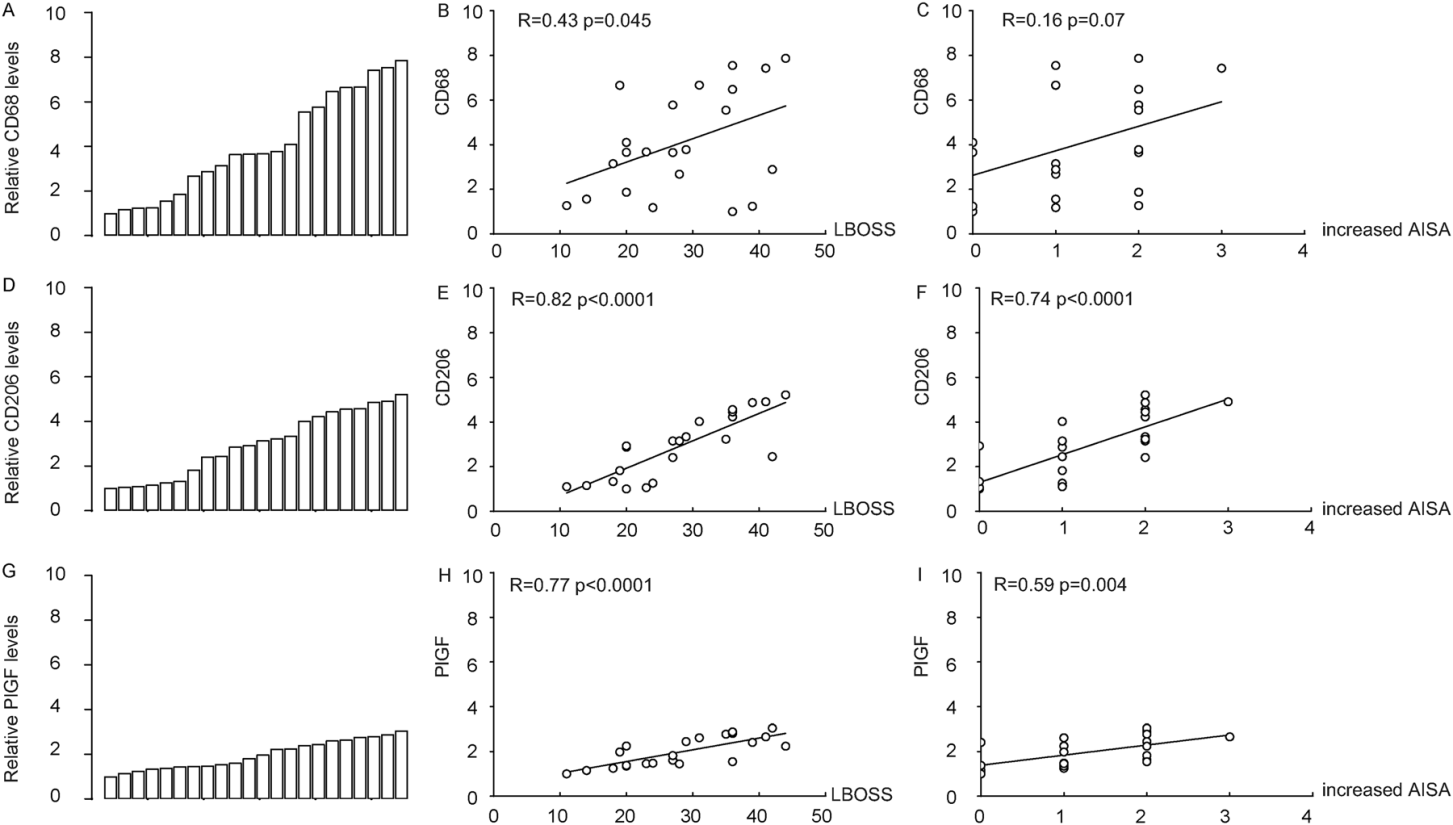
Patient data suggest a possible role of macrophages in the recovery of the internal fusion after thoracolumbar fracture. **A**–**F** Some patients with a diagnosis of non-pathological thoracolumbar burst fracture in the T12-L3 range who have received an internal fixation treatment were selected in a clinical and radiographic review using a Low Back Outcome Score System (LBOSS) and the changes in the grade of AISA at post-operation compared to pre-operation. The biopsy at the injured regional muscle was used to assess the levels of CD68, CD206, and PlGF. **A** RT-qPCR for CD68. **B** Correlaton between LBOSS and CD68 levels. **C** Correlaton between AISA changes and CD68 levels. **D** RT-qPCR for CD206. **E** Correlaton between LBOSS and CD206 levels. **F** Correlaton between AISA changes and CD206 levels. **G** RT-qPCR for PlGF. **H** Correlaton between LBOSS and PlGF levels. **I** Correlaton between AISA changes and PlGF levels. *N* = 22.

Recent studies suggest a crosstalk between MSCs and macrophage in regulation of tissue repair and regeneration, and PlGF is likely a key factor to regulate macrophage recruitment, differentation and polarization. We thus studies the expression of macrophage-specific and macrophage-differentiation-specific markers to see whether it may be related to the outcome of the internal fusion of the patients. Some patients with a diagnosis of non-pathological thoracolumbar burst fracture in the T12-L3 range (kyphos was above 20° and/or anterior body collapse was >50%) who have received an internal fixation treatment at our hospital between 2015 and 2018 were selected in a clinical and radiographic review using a Low Back Outcome Score System (LBOSS) adapted from a previous publication^36^, shown in Table 1, and using an ASIA grade to assess the nerve injury levels both pre-operatively and post-operatively. The biopsy at the injured regional muscle was used to assess the levels of macrophage infiltration by CD68, a pan-macrophage marker, the levels of macrophage differentiation by CD206, a M2 or anti-inflammatory macrophage marker, and the levels of PlGF, a newly determined trigger for macrophage recruitment and differentiation. CD68 levels were determined by RT-qPCR (Fig. 1A), which showed strong correlation with the LBOSS of the patients (*p* < 0.05; patients with better recovery appeared to have high macrophages at the injured/fusion site; Fig. 1B). The correlation between the CD68 levels with the AISA changes (every level improvement at post-operation compared to pre-operation is numbered 1, e.g., no improvement is 0; B to C is 1; A to C is 2) was also analyzed, showing a trend but not significant correlation (*p* = 0.07, Fig. 1C). CD206 levels were determined by RT-qPCR (Fig. 1D), which showed very strong correlation with the LBOSS of the patients (*p* < 0.001; patients with better recovery appeared to have higher M2 macrophages at the injured/fusion site; Fig. 1E) and the AISA changes (Fig. 1F). PlGF levels were determined by RT-qPCR (Fig. 1G), which also showed very strong correlation with the LBOSS of the patients (*p* < 0.001; patients with better recovery appeared to have higher PlGF levels at the injured/fusion site; Fig. 1H) and the AISA changes (Fig. 1I). Together, these data suggest a possible positive effect of macrophages in the internal fusion of the vertebraes, which inspired us to assess it in the mouse model.

**Table 1:** Low Back Outcome Score System for patients (LBOSS-adapted*).

Factor	Outcome	Points
Visual analogue scale of pain	7–10	0
	4–6	4
	0–3	8
Employment	Unemployed	0
	Part-time	4
	Full-time	8
Heavy social activities (sports, etc)	None	0
	Reduced	3
	Modestly reduced	6
	Back to previous level	9
Resting	More than half day	0
	Less than half day	2
	Little rest	4
	No need to rest	6
Live treatment	More than once per day	0
	Nearly everyday	2
	Infrequently	4
	Never	6
Sex life	Severely affected	0
	Modestly or little affected	3
	unaffected	6
Sleeping, walking and other regular activities	Severely affected	0
	Modestly or little affected	3
	unaffected	6

*Range: 0–49; outcome: poor → good.

### Generation of MSCs expressing PlGF

**Fig. 2:**
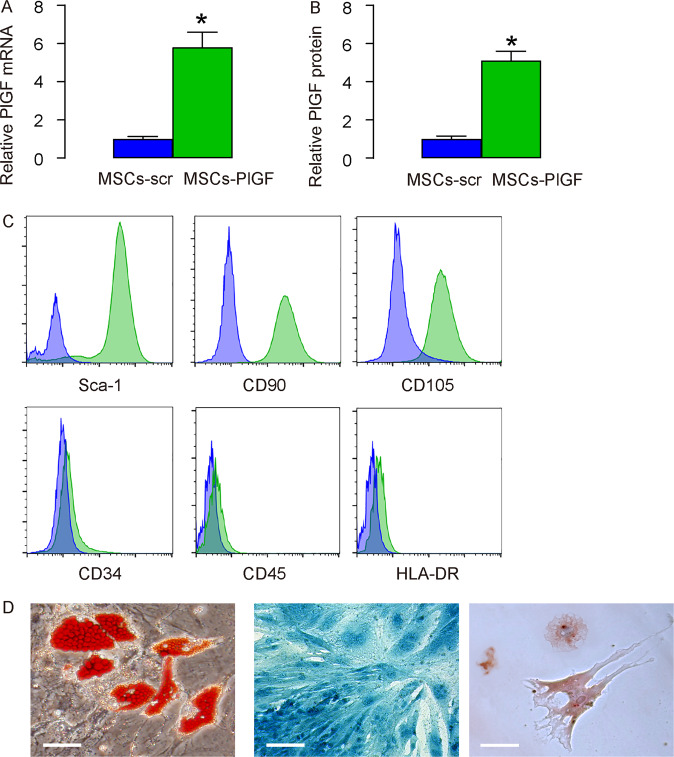
Generation of MSCs expressing PlGF. We generated MSCs that overexpress PlGF (MSCs-PlGF), and MSCs that express a scramble sequence as a control (MSCs-scr). **A**–**B** PlGF levels by RT-qPCR (**A**), and by ELISA (**B**). **C** Flow cytometry for surface markers in MSCs-PlGF. **D** Oil red O staining to evaluate adipogenic induction (left panel), alcian blue staining to evaluate chondrogenetic induction (middle panel), and Von kossa staining to evaluate osteogenic induction (right panel). *p < 0.05. *N* = 5. Scale bars are 50 µm.

In order to assess the effects of MSCs on macrophage recruitment and differentation during spinal fusion, especially through PlGF, we generated MSCs that overexpress PlGF (MSCs-PlGF), which could increase their potential to attract and polarize macrophages, as macrophages express the unique receptor, VEGF receptor 1, for PlGF. The increased PlGF in MSCs-PlGF, compared to control MSCs-scr, was confirmed by RT-qPCR (Fig. 2A), and by ELISA (Fig. 2B). The preservation of MSC characteristic by MSCs-PlGF was confirmed by expression of surface markers (Fig. 2C), and by maintanence of the differentating potentials (Fig. 2D).

### PlGF increases macrophage migratory potential and M2-differentiation by MSCs

**Fig. 3:**
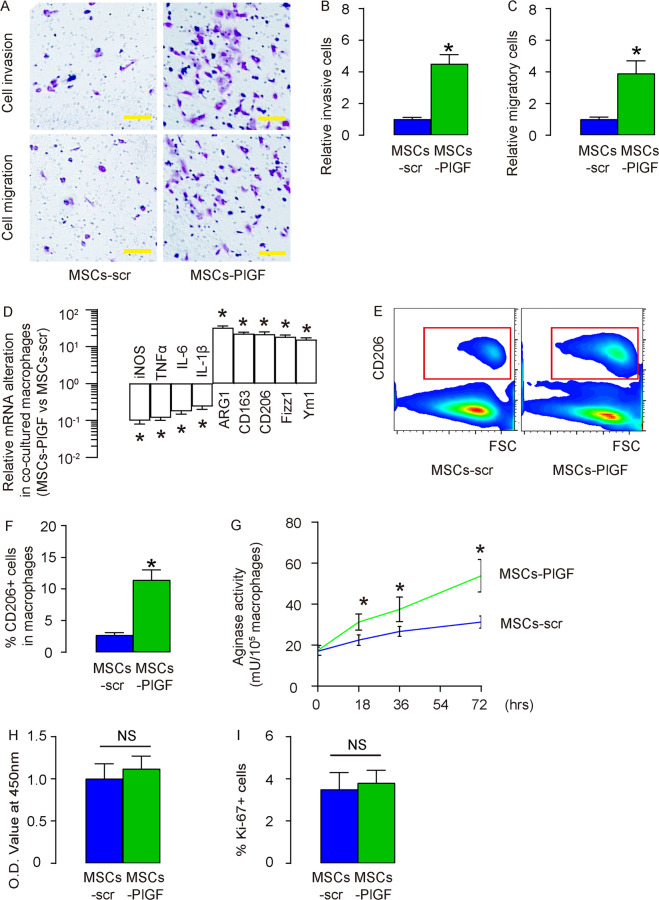
PlGF increases macrophage migratory potential and M2-differentiation by MSCs. **A**–**C** Transwell cell invasion assay and cell migraton assay, shown by representatitve images (**A**), and by quantification of invasive cells (**B**) and quantification of migratory cells (**C**). **D** RT-qPCR for genes in macrophages co-cultured with MSCs-PlGF versus macrophages co-cultured with MSCs-scr. **E**–**F** Flow cytometry for CD206 in macrophages co-cultured with MSCs-PlGF versus macrophages co-cultured with MSCs-scr, shown by representative flow charts (**E**) and by quantification (**F**). **G** Arginase activity assay. **H** Quantification of cell number of a CCK-8 assay. **I** Quantification of Ki-67+ cells by flow cytometry. *p < 0.05. NS non-significant. *N* = 5. Scale bars are 50 µm.

Next, we assessed the effects of expression of PlGF in MSCs on macrophages in a co-culture system. We found that PlGF expression in MSCs significantly increased the invasion and migration of co-cultured macrophages (Fig. 3A–C). Moreover, PlGF expression in MSCs significantly decreased the expression of M1/proinflammatory genes (iNOS, TNFα, IL-6 and IL-1β) and significantly increased the expression of M2/anti-inflammatory genes (Arginase 1 (ARG1), CD163, CD206, Fizz1 and Ym1) in the co-cultured macrophages (Fig. 3D). The PlGF-induced M2-like differentation of the co-cultured macrophages was furhter confirmed by analysis on M2 surface marker CD206 (Fig. 3E–F) and by increases in arginase activity (Fig. 3G). However, the total macrophage number in the co-cultured system was not significantly altered (Fig. 3G–I). Together, these data suggest that PlGF in MSCs increases macrophage migratory potential and M2-differentiation without altering macrophage growth.

### PlGF expression in MSCs promote spinal fusion in vivo

**Fig. 4:**
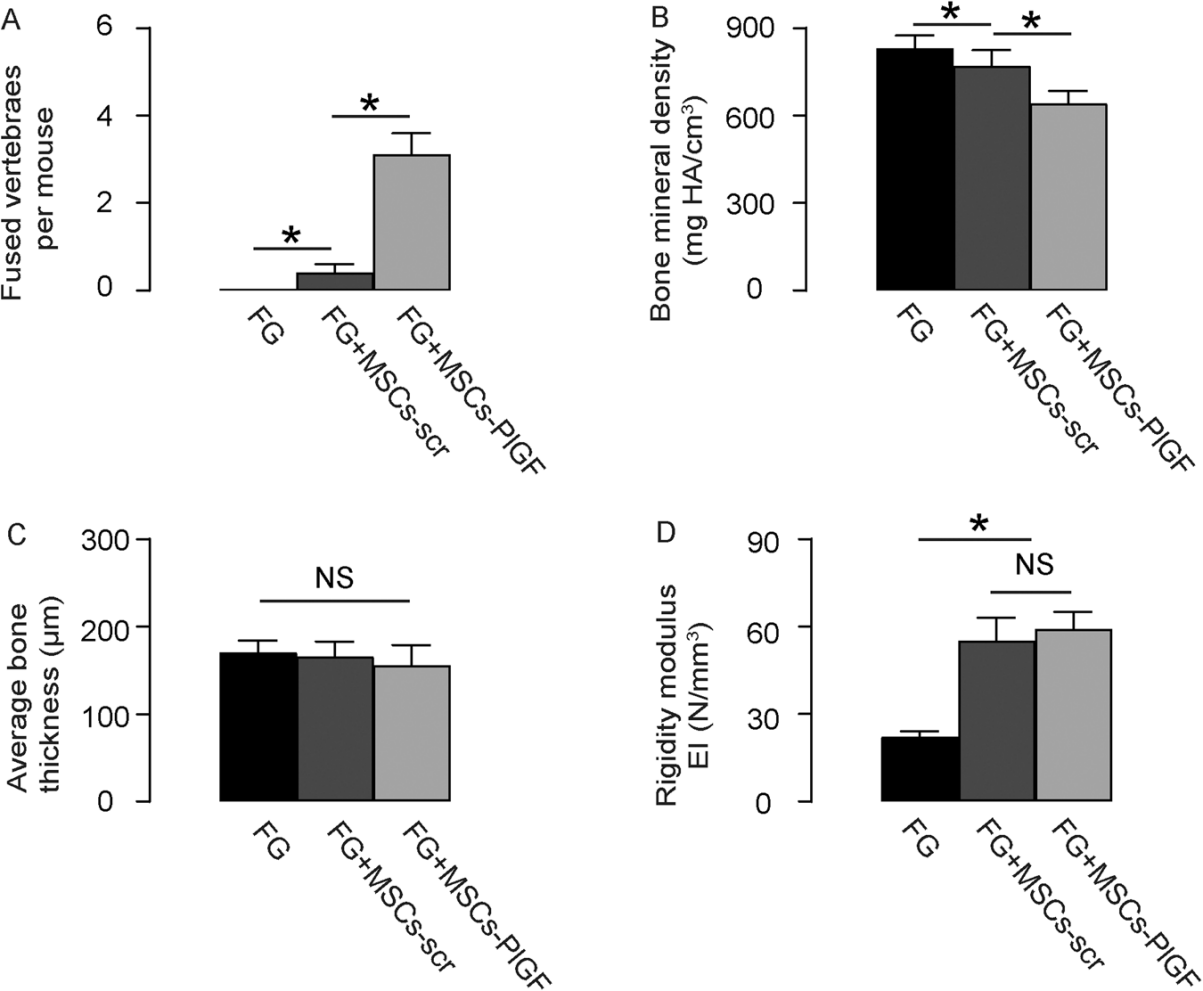
PlGF expression in MSCs promote spinal fusion in vivo. The effects of PlGF expression in MSCs on spinal fusion were tested in a mouse model. The mice were assigned into 3 groups of 8 each: Group 1: mice received injection of MSCs-free FG; Group 2: mice received injection of FG containing MSCs-scr; Group 3: mice received injection of FG containing MSCs-PlGF. All the mice were kept for 6 weeks before analysis. **A** Quantifiation of spinal fusion by fused vertebrae number. **B** Bone mineral density. **C** Average bone thickness. **D** Bone rigidity by EI. *p < 0.05. NS non-significant. *N* = 8.

The effects of PlGF expression in MSCs on spinal fusion were tested in a mouse model. The mice were assigned into 3 groups of 8 each: Group 1: mice received injection of MSCs-free FG; Group 2: mice received injection of FG containing MSCs-scr; Group 3: mice received injection of FG containing MSCs-PlGF. All the mice were kept for 6 weeks before analysis. We found that FG alone did not induce spinal fusion, while MSCs in FG induced a few spinal fusion, but MSCs-PlGF in FG induced significanly more spinal fusion (Fig. 4A). Bone mineral density was quantified, showing the highest in FG, while reduced in FG + MSCs-scr and further reduced in FG + MSCs-PlGF (Fig. 4B), which was consistent with the new bone formation (Fig. 4A). While average bone thickness among 3 groups were not significantly different (Fig. 4C), the spinal regidity between FG + MSCs-scr and FG + MSCs-PlGF groups was not significantly different (Fig. 4D), suggesting that the promotion of vertebrae fusion is not compensated by altering spinal regidity. Thus, PlGF expression in MSCs promote spinal fusion in vivo.

### PlGF expression in MSCs promote spinal fusion through macrophages

**Fig. 5:**
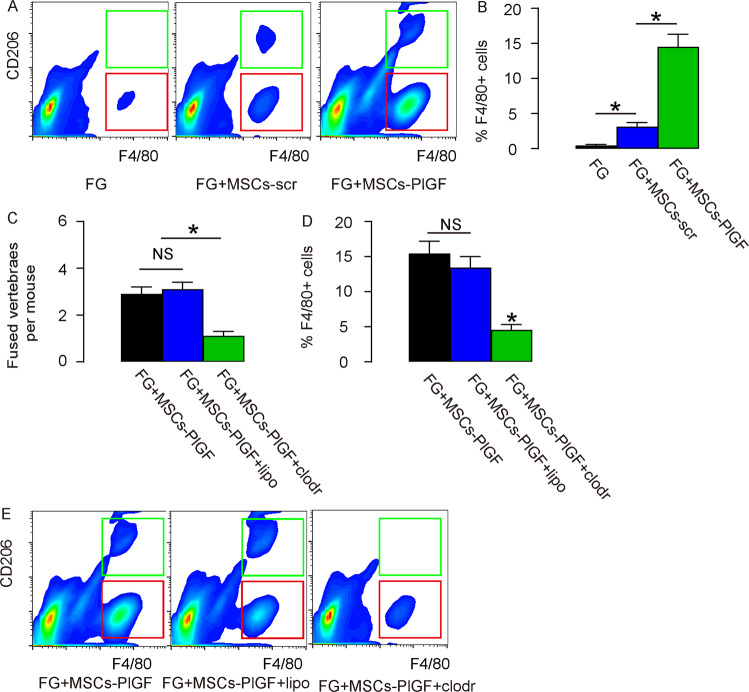
PlGF expression in MSCs promote spinal fusion through macrophages. **A**, **B** Flow cytometry analysis of the macrophage number in the fusion site, shown by representative flow charts (**A**) and by quantification (**B**). **C**–**E** In order to assess whether the recruitment of macrophages is the reason for the enhancement of spinal fusion by by PlGF-expressing MSCs, macrophages were chemically depleted by clodronate. For this experiment, the mice were assigned into 3 groups of 8 each: Group 1: mice received injection of FG containing MSCs-PlGF; Group 2: mice received injection of FG containing MSCs-PlGF and a weekly intravenous injection of liposome, the control for clodronate, till the end of the 6-weeks followup; Group 3: mice received injection of FG containing MSCs-PlGF and a weekly intravenous injection of clodronate to deplete macrophages. **C** Quantifiation of spinal fusion by fused vertebrae number. **D**–**E** Flow cytometry analysis of the macrophage number in the fusion site, shown by quantification (**D**), and by representative flow charts (**E**). *p < 0.05. NS non-significant. *N* = 8.

We examined the mechanisms underlying the enhancement of spinal fusion by PlGF-expressing MSCs. Analysis of the macrophage number in the fusion site showed signficantly increases in F4/80+ macrophages in FG + MSCs-scr mice, compared to FG mice, and this increase in macrophages further increased in the FG + MSCs-PlGF mice, shown by representative flow charts (Fig. 5A) and by quantification (Fig. 5B). In order to assess whether the recruitment of macrophages is the reason for the enhancement of spinal fusion by by PlGF-expressing MSCs, we performed another experiment, in which macrophages were chemically depleted by clodronate^39^. For this experiment, the mice were assigned into 3 groups of 8 each: Group 1: mice received injection of FG containing MSCs-PlGF; Group 2: mice received injection of FG containing MSCs-PlGF and a weekly intravenous injection of liposome, the control for clodronate, till the end of the 6-weeks followup; Group 3: mice received injection of FG containing MSCs-PlGF and a weekly intravenous injection of clodronate to deplete macrophages. We found that macrophage-depletion by clodronate abolished the effects of PlGF in MSCs on spinal fusion (Fig. 5C), and the macrophage depletion in clodronate-treated mice was confirmed by flow cytometry (Fig. 5D–E). Thus, PlGF expression in MSCs promote spinal fusion through macrophages.

## Discussion

Previous studies have demonstrated a seminal role of macrophages in the repair of the injured spinal cord^40^. These macrophages are mainly derived from circulating monocytes from bone marrow and spleen^40^. The injured tissue secretes cytokines and chemokines into circulation as chemoattractants for recruiting these monocytes into the site where they differentiate into macrophages to perform multiple functions involved in the wound healing process^41^. Although these studies have well defined a pivotal role of macrophages in the spinal repair, a similar role of macrophages in the MSCs-mediated spinal fusion has not been analyzed.

The total macrophages are contributed by M1 and M2 macrophages. Since we detected a strong correlation between LBOSS/AISA with the M2 marker CD206, but only a modest or no correlation between the total macrophage marker CD68 (M1 + M2) and LBOSS or AISA, respectively, which may be due to the much more important effect of M2 macrophages than M1 macrophages. M2 macrophages are the macrophage subtypes associated with tissue repair and regeneration through their production and secretion of a number of trophic cytokines. Moreover, the strong correlation between PlGF and the recovery suggests PlGF as a key factor to recruit macrophages, as reported by many studies in other area^29,31–34,42–46^. On the other hand, presence of M1 macrophages could have neutral or even negative effects on LBOSS, while the difference in M1 macrophage number/ratio to total macrophages in addition to the number of M2 macrophages may cause the variations of LBOSS.

In our in vitro co-culture experiment, PlGF expression in MSCs significantly increased the invasion and migration of co-cultured macrophages and polarized macrophages to a M2-like phenotype, suggesting that PlGF has a dual role in the regulation of spinal fusion by macrophages. First, macrophages are recruited to the site through being attracted to PlGF through its expression of VEGFR1. Second, the signaling cascades downstream of PlGF/VEGFR1 regulatory axis may activate the differentiation process in macrophages, perhaps through STAT1/STAT3/STAT6 signaling as reported^47–49^, to promote the bone formation during spinal fusion.

In our in vivo experiment, the depletion of macrophages by clodronate was sufficient to antagonize the effects of overexpressing PlGF in MSCs, again supporting the hypothesis that PlGF expression in MSCs promotes spinal fusion by macrophages. Interestingly, the use of clodronate not only depleted most of macrophages, but also seemed to preferably deplete all M2 macrophages. This result may be resulting from the different properties of M1 and M2 macrophages in the phagocytosis of clodronate or from the difference in their survival potential after engulfing clodronate^39,50,51^. The effects of PlGF-expressing MSCs were completely abolished by use of clodronate, suggesting that the major effective target of PlGF should be macrophages, rather than endothelial cells, since they are the two major cell types that express the unique receptor for PlGF on site, and clodronate does not affect endothelial cells at all^30^.

To summarize, here using both in vitro and in vivo tools, we were able to demonstrate a critical role of macrophages in the spinal fusion enhanced by transplantation of MSCs. Moreover, we found that PlGF could be a promising target gene to manipulate for regulating macrophage recruitment and differentiation at the fusion site.
